# Testosterone Reduces Myelin Abnormalities in the Wobbler Mouse Model of Amyotrophic Lateral Sclerosis

**DOI:** 10.3390/biom14040428

**Published:** 2024-04-01

**Authors:** Ivan J. Esperante, Maria Meyer, Carolina Banzan, Maria Sol Kruse, Analia Lima, Paulina Roig, Rachida Guennoun, Michael Schumacher, Alejandro F. De Nicola, Maria Claudia Gonzalez Deniselle

**Affiliations:** 1 Laboratory of Neuroendocrine Biochemistry, Instituto de Biologia y Medicina Experimental, CONICET, Buenos Aires 1428, Argentina; esperanteivan@gmail.com (I.J.E.); mariameyer1981@gmail.com (M.M.); c.banzan@gmail.com (C.B.); alejandrodenicola@gmail.com (A.F.D.N.); 2 Laboratory of Neurobiology, Instituto de Biologia y Medicina Experimental, CONICET, Buenos Aires 1428, Argentina; sol.kruse@conicet.gov.ar; 3 U1195 INSERM and University Paris Sud: “Neuroprotective, Neuroregenerative and Remyelinating Small Molecules”, 94276 Kremlin-Bicêtre, France; rachida.guennoun@inserm.fr (R.G.); michael.schumacher@inserm.fr (M.S.); 4 Departamento de Bioquímica Humana, Facultad de Medicina, Universidad de Buenos Aires, Buenos Aires 1121, Argentina; 5 Departamento de Ciencias Fisiológicas, UA1, Facultad de Medicina, Universidad de Buenos Aires, Buenos Aires 1121, Argentina

**Keywords:** myelin, testosterone, androgens, aromatase, anastrozole, ALS, Wobbler mouse

## Abstract

Amyotrophic lateral sclerosis (ALS) is a fatal motoneuron degenerative disease that is associated with demyelination. The *Wobbler* (*WR*) mouse exhibits motoneuron degeneration, gliosis and myelin deterioration in the cervical spinal cord. Since male *WR*s display low testosterone (T) levels in the nervous system, we investigated if T modified myelin-relative parameters in *WR*s in the absence or presence of the aromatase inhibitor, anastrozole (A). We studied myelin by using luxol-fast-blue (LFB) staining, semithin sections, electron microscopy and myelin protein expression, density of IBA1^+^ microglia and mRNA expression of inflammatory factors, and the glutamatergic parameters glutamine synthetase (GS) and the transporter GLT1. Controls and *WR* + T showed higher LFB, MBP and PLP staining, lower g-ratios and compact myelin than *WR*s and *WR* + T + A, and groups showing the rupture of myelin lamellae. *WR*s showed increased IBA1^+^ cells and mRNA for CD11b and inflammatory factors (IL-18, TLR4, TNFαR_1_ and P_2_Y_12_R) vs. controls or *WR* + T. IBA1^+^ cells, and CD11b were not reduced in *WR* + T + A, but inflammatory factors’ mRNA remained low. A reduction of GS^+^ cells and GLT-1 immunoreactivity was observed in *WR*s and *WR* + T + A vs. controls and *WR* + T. Clinically, *WR* + T but not *WR* + T + A showed enhanced muscle mass, grip strength and reduced paw abnormalities. Therefore, T effects involve myelin protection, a finding of potential clinical translation.

## 1. Introduction

Myelin disorders are present in common damage to myelin sheaths and to axons in the brain and/or spinal cord. The most frequent disease in this group is Multiple Sclerosis (MS) considered to be of primary autoimmune and inflammatory origin although some variants suggest primary neurodegeneration [1,2]. Although amyotrophic lateral sclerosis (ALS) is not categorized as a demyelinating disease, it shows white matter demyelination. First described by Charcot in 1869, it is characterized by the death of motoneurons in the ventral horn and/or medulla oblongata and axonal loss in the lateral columns of the spinal cord, which show a “sclerotic” appearance caused by damage, loss and scarring of the myelin sheaths [3,4,5]. Therefore, therapies directed to preserve myelin or remyelinate axons should be considered for the treatment of ALS neuropathology, in addition to therapies aiming to protect motoneurons.

Interestingly, a dysfunctional endocrine system is present in ALS patients, resulting in increases of circulating cortisol and progesterone [6]. Working with the *Wobbler (WR*) mouse mutant [4], a model of human motoneuron diseases such as ALS, we have shown protective effects of progesterone, its metabolite allopregnanolone and the synthetic progestin, Nestorone, for damaged motoneurons. These steroids decrease oxidative stress, excitotoxicity, neuroinflammation and death-related molecules, but increase trophic factors and prevent hyperactivation of microglia and astrocytes [7,8]. In fact, inflammation of the spinal cord appears in the *WR* mouse early after birth, suggesting that proinflammatory factors play an important part in neuronal and myelin damage [9]. *WR* mice also show a pronounced hypogonadism, infertility and low levels of testosterone (T) in the brain, spinal cord and blood [10,11,12]. Interestingly, the neuropathology of *WR mice* bears striking similarities to that of the *SOD1^G93A^* rat model of familial ALS, including massive myelin degeneration in the spinal cord [13].

In addition to the mentioned effects of progestins, androgens also show protective effects in the nervous system. Androgens prevent neuronal death caused by the activation of astrocytes and microglia after injury, glucose deprivation and excitotoxicity [14]. In addition, they stimulate neuronal differentiation and neurite outgrowth and inhibit oxidative stress due to mitochondrial dysfunction [15,16]. Thus, published evidence supports the anti-inflammatory, immunomodulatory and beneficial neuronal effects of androgens away from classical reproductive effects.

Regarding androgen effects on axons, Pesaresi et al. demonstrate robust sex differences in the g-ratio, an index of optimal axonal myelination, with males showing higher values than female animals. The increased g-ratio in males depends on higher levels of axon diameter that are not accompanied by an increase in the thickness of the myelin sheaths [17]. The beneficial properties of androgens also apply to the genesis of myelin and myelin regeneration. Hussein et al. (2013) have shown that androgen exposure improves remyelination in the corpus callosum after prolonged cuprizone-induced demyelination and in cultured cerebellar slices after acute lysolecithin-induced demyelination [18]. Ghoumari et al. (2020) have recently summarized the regenerative potential of T under the same conditions and also in lysolechitin-induced demyelination and experimental autoimmune encephalomyelitis [19,20]. Both the androgen receptor (AR) and T aromatization into estrogens are considered important players of the remyelinating effects of androgen [15].

In humans with ALS, Weinert hypothesized that the disease may be caused by the lack or dysfunction of AR in motoneurons [21]. In an experimental model of familial ALS, the synthetic androgen, nandrolone, enhances neuromuscular function and preserves mitochondrial morphology [22] although opposite effects have also been reported [23]. It has been postulated that androgen protective effects on neurons may be due to crosstalk of AR with insulin growth factor1, although this interaction could obscure a direct participation of androgen effects [24]. In humans with MS and mice with experimental demyelination, Zahaf et al. (2023) have shown that androgens synergize with estrogens to recruit oligodendrocyte precursors to demyelinating lesions and that there are specific effects of androgens in the response of glial cells to myelin loss [25]. Additional studies reporting androgen effects on peripheral nerves which originated in Melcangi’s laboratory described androgen protection of peripheral myelin containing the peripheral glycoprotein P0 and the peripheral myelin protein 22 (PMP22) from damage inflicted by injury, aging and diabetes [26,27,28]. Therefore, both peripheral as well as central myelin proteins are regulated after treatment by T or its metabolites.

Former work from our laboratory has demonstrated several T effects in the *WR* mouse. Thus, treatment with T increases the enzyme, choline acetyltransferase, in motoneurons, decreases motoneuron vacuolation (a hallmark of the disease), decreases astrogliosis, microgliosis, oxidative and nitrosative stress, reduces proinflammatory factors and improves motor behavior and paw atrophy [29]. Thus, in the former study, we focused on androgen protective effects on spinal cord motoneurons and on reduction in reactive glial cells. In the present work, we studied several myelin-related parameters in *WR* mice with or without T treatment. To this end, we performed quantitative histochemistry of whole myelin, electron microscopy to asses myelin compaction and expression of myelin proteins. We also measured inflammatory mediators and molecules of the glutamate circuit that may impair myelination. The role of T-derived estrogens on myelination was also analyzed by exposing T-treated *WR* mice to the aromatase inhibitor, anastrozole (A).

## 2. Materials and Methods

### 2.1. Experimental Animals and Ethical Statement

Control and *WR* mice on an *NFR/NFR* background were raised at the animal room facilities of the Instituto de Biologia y Medicina Experimental. Two groups were identified by genotyping analysis, i.e., wild-type male control mice (*NFR/NFR*) and male *WR* mice (*wr*/*wr*) [11]. Mice were kept under standard conditions, i.e., controlled humidity and temperature (22 °C), lights on from 07:00 am to 07.00 pm and fed standard mice chow ad libitum. Two-month-old *WR*s were used at the symptomatic stage which showed tremor, flexion of proximal limbs, ambulatory difficulty, diminished muscle strength and lighter body weight compared to control mice [30]. *WR* mice sacrificed at this stage show about 25% vacuolated motoneurons, a hallmark of this disease. Sample size was calculated taking into account our previous experience depending on each technique and considering a confidence interval of 95% and a medium significant size effect [7,31]. The results shown correspond to the most representative experiment from 2 experiments performed at different times. In addition, astrogliosis and microgliosis were detected by staining for cell specific markers in both gray and white matters of the cervical spinal cord [32]. All procedures reported in this paper followed the guide for the Care and Use of Laboratory Animals (NIH Guide, Instituto de Biologia y Medicina Experimental Assurance Certificate #A5072-01) and were approved by the Institute’s Animal Care and Use Committee on 23 July 2021. The experiments are reported in accordance with the ARRIVE guidelines (www.nc3rs.org.uk).

### 2.2. Treatment with Testosterone and Anastrozole, an Aromatase Inhibitor

Male mice were randomly and blindly assigned to the treatment groups. In order to analyze the effect of androgen treatment and the cotreatment of T plus the aromatase inhibitor, A, in control mice, we evaluated the effect of T or T + A treatment in male controls (*NFR/NFR*) on body weight progression and the size of seminal vesicles. As shown previously, controls + T-treated mice [29] show no differences compared to T-free controls. A similar response was found in controls + T + A-treated mice. Therefore, the following experimental groups were prepared: (1) control (CTL; n = 15), (2) *WR* (n = 15), (3) *WR* + T (n = 15), (4) *WR* + T+ A (n = 15). Steroid delivery was performed by implantation of silastic tubes (Down Corning) filled with crystalline T (Sigma-Aldrich, St Louis, MO, USA) under the skin of the neck. Tubes measured 1.57 mm in inner diameter, 2.41 mm in outer diameter and 10 mm in length. Before s.c. implantation, tubes were immersed in 70% ethanol and then washed in sterile 0.9% NaCl solution. Silastic tubes were implanted in mice anesthetized with isoflurane (induction 4%; maintenance 1.5%; BAXTER Healthcare Corp., Deerfield, IL, USA) and remained in place for 60 days. The hormone delivery method produces physiological levels of T for at least 60 days as determined by gas chromatography/mass spectrometry in male mice [18,29] and promotes myelin formation in both brain and spinal cord in a mouse demyelination model [19]. Control and *WR*s not treated with T received s.c. empty silastic tubes for 60 days.

**Figure 1:**
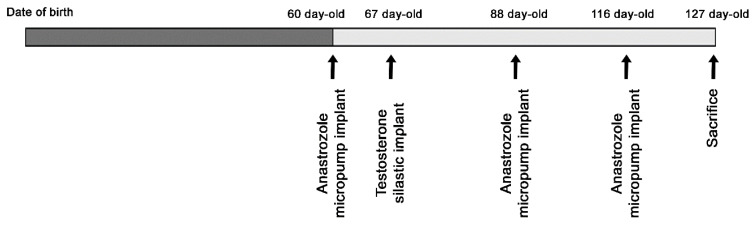
Time protocol for testosterone and anastrozole treatments in male Wobbler mice. Animals were implanted with a silastic tube filled with T at 67 days of age or a silastic tube filled with T at 67 days of age plus a micropump filled with anastrozole at 60, 88 and 116 days of age. Mice were killed at 127 days of age.

The aromatase inhibitor, A (gift from Gador Labs, Buenos Aires, Argentina) was dissolved in 10% dimethylsulfoxide (DMSO) and introduced into Alzet osmotic micropumps (model 1004-Alzet, Cupertino, CA, USA) showing a delivery rate of 1 mg/kg/day. Micropumps were implanted s.c. starting 1 week before T treatment and remained in place for 28 days, the period during which the delivery lasts. Then, two micropumps were reimplanted consecutively to complete the time of treatment (Figure 1).

### 2.3. Semithin Sections and Electron Microscopy Studies

Sixty days after silastic tube implantation, 12 mice (controls n = 3, untreated *WR*s n = 3, *WR* + T n = 3 or *WR* + T + A n = 3) were anesthetized with a mixture of 6 mg/kg xylazine (Bayer, Argentina) and 75 mg/kg ketamine (Holliday-Scott, Béccar, Argentina) and intracardially perfused with ice-cold 4% paraformaldehyde in 0.1 M phosphate buffer pH 7.4 for electron microscopy. The cervical region of the spinal cord was dissected, the ventral region was cut into 2–3 mm length sections and immersed for 2 h in glutaraldehyde 2.5% in 0.1 M phosphate buffer at a pH of 7.4. After washing in the same buffer solution, sections containing the ventrolateral funiculus were postfixed for 60 min in 1% OsO_4_ in 0.1 M phosphate buffer at a pH of 7.4. A final staining step was performed with 1% uranyl acetate. Afterwards, tissue sections were dehydrated and flat-embedded in Durcupan (Fluka Chem. AG, Göteborg, Sweden) for 72 h at 60 °C. Semithin (0.5 μm) and ultrathin sections (60–70 nm) were obtained using an ultramicrotome (Reichert Jung Ultracut E). Ultrathin sections were stained with lead citrate and examined at several magnifications (12,000×, 50,000× and 250,000×, respectively). Images were photographed using a Gatan W 10,000 camera connected to a Zeiss 109 electron microscope (Oberkochen, Germany). Sectioning and staining procedures were carried out at the Dept. of Histology, Faculty of Medicine, University of Buenos Aires. In addition to electron microscopy, we analyzed the density and morphological appearance of axons in the 4 experimental groups. For this purpose, semithin sections were stained with 2% toluidine blue for light microscopy observations and quantitation of the myelin thickness/axon diameter ratio [33,34]. G-ratios are presented as the axon diameter/total diameter of the axon plus the myelin sheath. Axon diameter and myelin thickness were calculated from the measured area based on the assumption of circularity using Fiji/ImageJ v1.52a (diameter = 2 × √[area/π]), with a minimum of 100 axons analyzed per animal (3 animals/group). Images of semithin sections were acquired using an Axiophot Zeiss light microscope and mentioned parameters were measured using a Fiji through the semi-automated quantification of axon diameters and g-ratio via MyelTracer software v1.3.1 [34]. As an additional measure, we conducted correlative analysis of myelin thickness (in µm) vs. axon diameter (in µm) and the differences between slopes were analyzed according to Dillenburg et al. [35].

### 2.4. Samples for Histological and Immunohistological Analyses

Anesthetized mice were intracardially perfused with ice-cold 4% paraformaldehyde (PFA) in 0.1 M sodium phosphate buffer at a pH of 7.4. Cervical spinal cords were dissected and post-fixed in the same fixative for 2.5 h. Then, C_2_–C_4_ segments were embedded in paraffin or C_4_–C_6_ segments were frozen on dry ice after cryoprotection in 20% sucrose overnight. Sections measuring 5 or 16 μm were cut using a microtome or a cryostat, respectively, and laid on microscope slides.

#### 2.4.1. Luxol Fast Blue (LFB) Staining of Whole Myelin

LFB staining was carried out according to Kim et al., 2006 [36]. Briefly, 5 μm paraffin sections were treated with 95% ethanol and left in LFB solution (0.1 g LFB, 0.5 ml 10% acetic acid, 95% ethanol to 100 mL) at 60 °C for 18 h. After several washes, sections were immersed in lithium carbonate, then 70% ethanol, rinsed in distilled water, dried and mounted with Permount. The percentage of LFB staining area of the ventrolateral funiculus above an established threshold was determined using computerized image analysis as described before [37].

#### 2.4.2. Immunohistochemistry of Myelin Basic Protein (MBP) and Proteolipid Protein (PLP)

Paraffin sections measuring 5 μm from cervical spinal cords of the different groups of mice were deparaffinized and treated with 0.3% hydrogen peroxide in methanol for 30 min to block endogenous peroxidase. Immunocytochemistry was carried out using two different primary antibodies diluted in PBS containing 1% goat serum: a rabbit anti-MBP primary antibody (1:500, Code AO623, Dako Cytomation, Carpinteria, CA, USA) and rat anti-PLP AA3 antibody (1:250, Millipore Sigma-Aldrich, St Louis, MO, USA). After overnight incubation at 4 °C and several washes with PBS, sections were incubated with either a 1/200 dilution of a biotinylated goat anti-rabbit IgG secondary antibody (Vector Labs, Newark, CA, USA) for 1 h at 22 °C, then with avidin-biotin-peroxidase (ABC) complex for 30 min (ABC kit Vector Labs, Newark, CA, USA) and revealed with 0.5 mg/mL diaminobenzidine tetrachloride (Sigma-Aldrich, St Louis, MO, USA) in the presence of 0.01% H_2_O_2_ for 7 min in the dark. The sections were given a final rinse in PBS, dehydrated in graded ethanol and xylene, and mounted with Permount [38].

For quantitative evaluation, MBP or PLP immunoreactive areas were delimited in the ventrolateral funiculus of the cervical spinal cord via computerized image analysis using Optimas VI software v6.5 [37]. The percentage of immunoreactive MBP or PLP area of each spinal cord section was quantified and expressed as a percentage of the total surface area of white matter sampled [37]. For quantitation of changes, comparative sections of the 4 experimental groups were averaged to obtain an independent figure for each animal (n = 4–8 animals per experimental group).

#### 2.4.3. Immunohistochemical Analysis of Glutamine Synthetase (GS) and Its Colocalization with Glial Markers

Literature data support that both astrocytes and oligodendrocytes express GS [39,40]. To determine the effects of T treatment on GS protein expression and its localization, we investigated the density of GS immunostained cells/unit area (A) and also performed double labeled colocalization of GS with the astrocyte marker, GFAP, with the microglial marker IBA1 and with the oligodendrocyte marker CNPase (B). (A) For quantitation of GS ^+^ cells, 5 μm paraffin sections from cervical spinal cord were blocked with mouse IgG blocking reagent (Vector M.O.M. Immunodetection Kit) and then incubated with a 1/200 dilution of monoclonal mouse anti-GS (BD Biosciences Cat.# 610517, RRID:AB_397879). We then used a 1/200 dilution of a goat antimouse biotinylated IgG (Vector Labs, Newark, CA, USA) and processed it according to the instructions of the ABC kit (Vector Labs, Newark, CA, USA). The peroxidase activity was revealed using diaminobenzidine tetrachloride (DAB, 0.25 mg/mL, SIGMA, St. Louis, MO, USA) in 0.01% H_2_O_2_ for 7 min in the dark. After a final rinse in PBS, sections were dehydrated in ethanol and xylene, and covered with mounting media (Permount).

(B) For astrocyte colocalization, after GS immunostaining revealed with a secondary donkey anti-mouse Alexa Red 555 (GS), a polyclonal rabbit anti-GFAP (1:250 dilution, Dako, Glostrup, Denmark) was incubated and, after several washes in PBS, followed by a secondary donkey anti-rabbit Alexa Green 488. To determine microglial colocalization, 16 μm cryostat sections were used for GS immunostaining, followed by a polyclonal rabbit anti-IBA1 (1/1000 dilution, Wako, Richmont, VA, USA) antibody for 3 days that was revealed with a secondary donkey anti-rabbit Alexa 647 antibody. For oligodendrocyte colocalization, paraffin sections subjected to antigen retrieval with HCl and boric acid followed by citrate buffer (pH = 6) were incubated with the rabbit polyclonal antibody, anti-CNPase (1/200, Sigma-Aldrich, St Louis, MO, USA) for 2 days at 4 °C followed by incubation with a secondary donkey anti-rabbit Alexa Green 488 (1/1000, Molecular Probes, Molecular Probes, Eugene, OR, USA) for 1 h at room temperature. After several washes in PBS, the sections were incubated with the GS antibody overnight at 4 °C, followed by the secondary donkey anti-mouse Alexa Red 555 antibody (1/500). Dual-labeled immunofluorescence microscopy sections for GS^+^/GFAP^+^ and GS^+^/IBA1^+^ were analyzed using an Olympus IX83 inverted microscope equipped with a disk-spinning unit (Olympus Corporation, Shinjuku City, Tokyo Japan) and images were acquired using Cell Sens Dimensions software v 1.18 from Olympus and photographed using a Hamamatsu Orca Flash 4.0 monochromatic camera (Hamamatsu Photonics K.K, Hamamatsu, Shizuoka, Japan). GS^+^/CNPase^+^ sections were visualized and images acquired using a laser scanning confocal microscope (Confocal Zeiss LSM880—Airyscan-Elyra, Zen Black 2.3) with a high-sensitivity system with a detector (Airyscan).

#### 2.4.4. Immunohistological Localization of Glutamate Transporter-1 (GLT-1)

Paraffin sections were blocked with 1% H_2_O_2_ followed by 10% goat serum in PBS and exposed to a primary guinea pig GLT-1 polyclonal antibody (1:1000, cat. #AB 1783 Millipore, St Louis, MO, USA). Then, sections were incubated with a secondary anti-guinea pig antibody from Vector (1:200). Sections were then processed according to the ABC kit instructions (Vector Labs, Newark, CA, USA). GLT-1 immunoreactivity (IR) was expressed as the percentage of GLT-1 reactive area of the ventral horn.

#### 2.4.5. Immunohistological Localization of the Oligodendrocyte Marker, CC1

Paraffin sections measuring 5 μm were blocked with 3% horse serum, and incubated with the monoclonal CC1 antibody (anti-CC1 OP80, Calbiochem, La Jolla, CA, USA) at a 1/100 dilution in 2% horse serum for 48 h at 4 °C. Then, sections were rinsed and incubated with the horse anti-mouse secondary antibody conjugated to Alexa Fluor 488 (1/500, Invitrogen, Molecular Probes, Eugene, OR, USA) for 1 h at room temperature. Next, sections were rinsed in PBS 0.1% Triton X-100 and mounted with Fluoromont G. Non-specific staining was performed in sections without the anti-CC1 primary antibody. Cell positive immunofluorescence was analyzed using an Olympus IX83 inverted microscope equipped with a disk-spinning unit (Olympus Corporation, Shinjuku City, Tokyo, Japan) and images were acquired using Cell Sens Dimensions software v1.18 from Olympus and photographed using a Hamamatsu Orca Flash 4.0 monochromatic camera (Hamamatsu Photonics K.K, Japan). The number of CC1+ cells was analyzed using the Fiji/Image J software v1.52a (NIH, MD, USA) and cell density was quantified as the number of CC1+ cells/100,000 μm^2^ of the ventrolateral funiculus and expressed as a percentage of control positive cells.

### 2.5. Quantitative RT-PCR Analysis of mRNA Expression of Myelin Proteins, Inflammatory Factors and Glutamate Transporter

The response of *WR* mice spinal cord to hormone treatment and to aromatase inhibitors was also determined at the transcriptional level. We thus measured the mRNAs for (1) the myelin proteins: MBP, PLP and myelin oligodendrocyte glycoprotein (MOG), (2) proinflammatory factors: CD11b, TLR4, TNFα receptor_1_ (TNFαR_1_), IL-18 and P_2_Y_12_R and glutamate metabolism: GLT-1. All mRNA levels were measured via real-time PCR using previously published procedures [41,42]. Sequences of primers are shown in Table 1, including cyclophilin as the housekeeping gene. In brief, total RNA was extracted from cervical spinal cord with Trizol (cat.#15596026, Life Technologies-Invitrogen, Carlsbad, CA, USA), and residual DNA was hydrolyzed with DNase1 (cat.# EC 3.1.21.1, Promega, Madison, WI, USA). For PCR amplification of DNA templates, we used a M1705 MMLV reverse transcriptase (cat# EC 2.2.2.49; Promega, Madison, WI, USA) in the presence of random hexamer primers. A real-time Step-one Plus Sequence Detection System (Applied Biosystems, Foster City, CA, USA) was used to establish gene expression profiles, and mRNA expression was analyzed using the 2^−ΔΔct^ method [43]. The results were expressed as fold induction over steroid untreated control *NFR/NFR* mice.

### 2.6. Clinical Assessments

The clinical effect of T alone and T + A cotreatment was studied in four different ways. First, the examination of the body weight was performed weekly. Second was by measurement of the biceps weight; third was by subjecting mice to a grip strength test [31]. In this test, empty-silastic-treated *WR*s, *WR* + T and *WR* + T + A were placed on a vertical grid and the time spent on the grid until they fell down was recorded in seconds. The time that control mice stayed on the grid could not be determined because they were able to climb to the top of the grid, while Wobblers were unable to climb against gravity. The trial was determined 3 times per animal and averaged. This determination was made weekly from the beginning (week 0: pre-treatment) until the end of the treatment (week 8). Fourth, we also evaluated the overall clinical score by assessing the grade of the deformity of the forelimbs and the eye score in the three groups of *WR*s. The atrophy of forelimbs and eye alteration were scored following Mitsumoto et al., 1994. Deformity of forelimbs included: (1) paw atrophy; (2) flexed digits; (3) flexed wrist; and (4) complete flexion of the limb over the chest. Eye condition included: 0—normal; 1—attached exudate; 2—narrowed palpebral fissure (unilateral); 3—narrowed palpebral fissures (bilateral); and 4—clogged eye (single or both) [44,45].

### 2.7. Statistical Analysis

All data were reported as mean ± SD. The analysis of body weight and vertical grid was expressed as mean ± SEM. The Shapiro–Wilk test was used to explore the normality of variables and the Levene test was used to detect homogeneous variances. Group differences were analyzed by one- or two-way ANOVA followed by the Tukey post hoc test to assess pairwise comparisons between group means. In order to analyze the progression of body weight and the performance in the vertical grid test, we analyzed data using repeated measures two-way ANOVA. Statistical analysis was performed using Prism 9 GraphPad software (San Diego, CA, USA). A *p* value < 0.05 was considered statistically significant.

## 3. Results

### 3.1. LFB Histochemistry Shows Preservation of Total Myelin in Testosterone-Treated WR Mice

**Figure 2:**
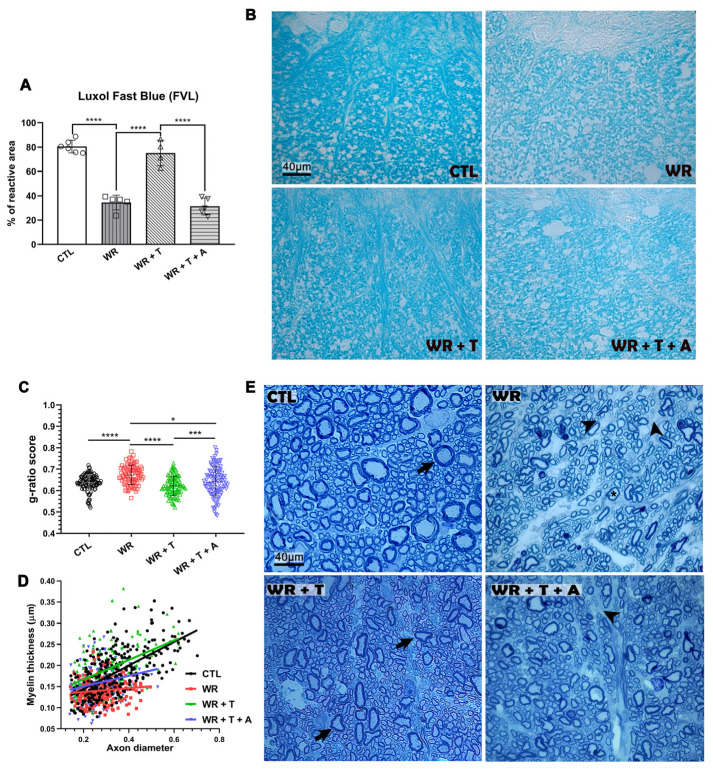
Effect of testosterone and anastrozole on myelination in Wobbler mice. (**A**): LFB staining of whole myelin in the spinal cord. *WR* mice showed decreased LFB reactive area vs. controls (*p* < 0.0001). In *WR* + T, LFB reactive area returned to control levels (*WR* + T vs. CTL, NS; *WR* + T vs. *WR p* < 0.0001). The *WR* T + A group showed a pronounced decrease in LFB reactive area (*p* < 0.0001 vs. *WR* + T; NS vs. *WR*). (**B**): Images of LFB staining in the ventrolateral funiculus of a CTL, *WR*, *WR* + T and a *WR* + T + A groups. Scale bar: 40 μm. (**C**): Statistical comparison of g-ratios in the four experimental groups. G-ratios were higher in *WR* mice vs. control mice (**** *p* < 0.0001), WR+T+A (* *p* < 0.05) and T treatment decreased this ratio vs. untreated *WR* mice (**** *p* < 0.0001). The graph also shows that inhibition of aromatase in the *WR* + T group increased the g-ratio (*** *p* < 0.001 vs. *WR* + T). (**D**): Plot of the relationship between the axon diameter and the myelin thickness. (**E**): Toluidine blue staining of semithin sections in the four experimental groups. Loss of myelinated axons (arrowhead) and large-caliber axons with abnormally thin myelin sheaths (asterisk) are clearly observed in the *WR* mouse compared to strong myelinated axons in control and *WR* + T (arrows). *WR* + T + A group showed a decreased density of myelinated axons (arrowhead). Scale bars: 40 μm. CTL: control, *WR*: Wobbler, T: testosterone, A: anastrozole.

The determination of myelin histochemical area in the ventrolateral funiculus using LFB showed significant group differences in the ANOVA test (F _(3,16)_ = 68.34, *p* < 0.0001). A multiple comparison test showed a 50% decrease in LFB staining in the ventrolateral funiculus of *WR* spinal cord (*p* < 0.0001 vs. control mice). In T-treated *WR*s, % LFB reactive area was significantly increased vs. empty-silastic-treated *WR*s (Figure 2A, *p* < 0.0001) reaching the levels for control mice. 

To determine if estrogen synthesis plays a role in T-induced remyelination, *WR*s received T plus the aromatase inhibitor, A (Figure 2A). Blockage of aromatase resulted in low levels of LFB staining area (*p* < 0.0001 vs. *WR*s + T) suggesting that T aromatization into estrogens contributes to myelin preservation. Figure 2B shows representative images of LFB staining in the ventrolateral funiculus.

### 3.2. Testosterone Restores the Thickness of Myelin Sheaths in Wobbler Mice

Toluidine blue staining of semithin sections was employed to discern myelination status and calculation of myelin thickness, axon diameter and the g-ratio (i.e., the ratio of the inner to outer diameter of a *myelinated* axon in the four experimental groups). G-ratio is widely used as a functional and structural index of myelin sheath thickness of individual axon fibers [35]. One-way ANOVA showed significant differences between groups (F_(3,394)_ = 16.08, *p* < 0.0001, Figure 2C). In particular, g-ratio scores were higher in *WR*s vs. control mice (*p* < 0.001). Instead, T treatment of the *WR* mice decreased g-ratios (*p* < 0.001 vs. untreated *WR*) but in *WR* + T + A, g-ratios increased to the levels of the untreated *WR* group (*p* < 0.001). Changes in g-ratios, therefore, underlined once more that demyelination of the *WR* group was restored by T treatment, and further suggested a role of aromatase in this event. To better assess the current differences in myelin and axons in the four experimental groups, we plotted myelin thickness (in µm) vs. axon diameter (in µm) following the procedure of Dillenburg et al. 2018 [35]. The graph in Figure 2D shows that the regression line for control mice (Y = 0.2774 × X + 0.08884) and for T-treated *WR* (Y = 0.2302 × X + 0.1208) were different from zero (*p* < 0.001 for both conditions). Instead, the regression line of steroid untreated *WR* was not significantly different from zero (y = 0.04479 × X + 0.11208, *p* > 0.05). The regression line for the *WR* + T + A group was also significant (y = 0.1287 × X + 0.11244, *p* > 0.05) although it was shorter than the lines of the control and *WR* + T groups. Figure 2E shows representative images of semithin sections from the ventrolateral funiculus of a control, *WR*, *WR* + T and *WR* + T + A. Control axons (CTL) show a predominant round morphology with compacted, well-preserved myelin sheaths. In comparison, *WR* mice showed small-caliber axons with very thin myelin sheaths, suggesting demyelination. Instead, the *WR* + T group showed myelinated axons of several sizes suggesting partial remyelination. The T effect was in part estrogen dependent, since blockage of aromatase with A showed a coexistence of myelinated axons with demyelinated axonal profiles (*WR* + T + A group in Figure 2E).

### 3.3. Testosterone Restores Axonal Myelination in WR Mice: Analysis by Using Electron Microscopy (EM)

**Figure 3:**
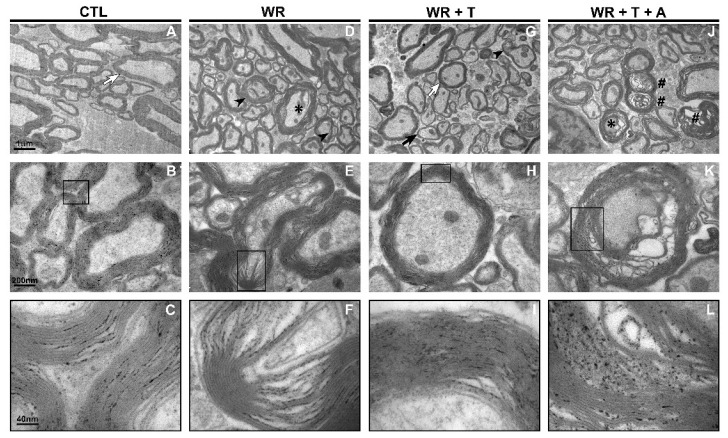
Effects of testosterone on axonal myelination and myelin compaction in Wobbler mice: digital Images of EM of the ventrolateral funiculus of the cervical spinal cord. (**A**–**C**): CTL mouse, (**D**–**F**): *WR* mouse, (**G**–**I**): *WR* + T mouse, (**J**–**L**): *WR* + T + A mouse. (**A**,**D**,**G**,**J**) are low power magnification images at 12,000× and (**B**,**E**,**H**,**K**) are high power magnification images at 50,000×, whereas (**C**,**F**,**I**,**L**) correspond to insets in (**B**,**E**,**H**,**K**), respectively, taken at 250,000×, scale bar: 40 nm. The EM photographs of CTL and *WR* + T mice showed axons surrounded by well-preserved myelin sheaths (white arrows in (**A**,**G**)). Small-caliber axons in the *WR* + T mouse suggest remyelination (black arrow, (**G**)) and few signs of myelin abnormalities with invagination of myelin lamellae (arrowhead, (**G**)). In contrast, images taken at both magnifications of untreated *WR* and *WR* + T + A mice showed irregularly shaped axons with dissolution and detachment of myelin lamellae (asterisks), invagination of myelin lamellae (arrowhead) and signs of axonal degeneration (hash symbol #). The insets in (**C**,**I**) depict the preservation of myelin sheath lamellae in the *WR* + T whereas those in (**F**,**L**) indicate myelin derangement in the *WR* and *WR* + T + A groups. Scale Bars: 1µm (upper), 200 nm (middle), 40 nm (bottom). CTL: control, *WR*: Wobbler, T: testosterone, A: anastrozole. EM: electron microscopy.

In the ventrolateral funiculus, control mice receiving empty silastic tubes showed thick-caliber myelinated axons, with well-compacted myelin sheaths and with a circular or elongated shape. Some mitochondrial and punctiform structures resembling microtubules were observed inside the axonal cytoskeleton (CTL-Figure 3A: low magnification, Figure 3B: high magnification, Figure 3C: inset from B). These images are in deep contrast with those from *WR* mice. The latter showed irregularly shaped axonal profiles, with detachment, dissolution and rupture of myelin lamellae (Figure 3D) and a few visible mitochondria and microtubules in the cytoskeleton. These images indicate axonal degeneration and demyelination (*WR*-Figure 3D: low magnification, Figure 3E: high magnification, Figure 3F: inset from E). In T-treated *WR* mice, circular and elongated axonal profiles reappear, with well-compacted myelin sheaths surrounding axons containing mitochondria (*WR* + T, Figure 3H). Moreover, the abundance of thinner myelinated axons in *WR* + T suggests reduced degeneration and enhanced remyelination (Figure 3 *WR* + T, G: low magnification, H: high magnification, I: inset from H). The percentage of axons with myelin abnormalities in the ventrolateral funiculus was 41.2 in *WR*, 24.4 in *WR* + T and 54.6 in *WR* + T + A inside an 800 μm^2^ field. Consistent with the results for semithin sections, androgen protective effects seemed in part to depend on estrogen synthesis. Thus, aromatase inhibition returned distorted axons, reduced myelin thickness with partial detachment and disorganization of myelin lamellae which had an onion-like appearance when examined by using EM (*WR* + T + A, Figure 3J: low magnification, K: high magnification, L: inset from K). Still, mitochondria were visible inside these pathological axons.

### 3.4. Testosterone Modulates the Expression of Myelin Proteins

The results obtained using LFB, semithin sections and EM indicated decreased thickness and compaction of myelin sheaths in *WR* mice. As dysregulation of myelin protein expression may compromise myelin formation and integrity, these data were complemented with assays of the expression of the major central myelin proteins MBP, PLP and MOG.

**Figure 4:**
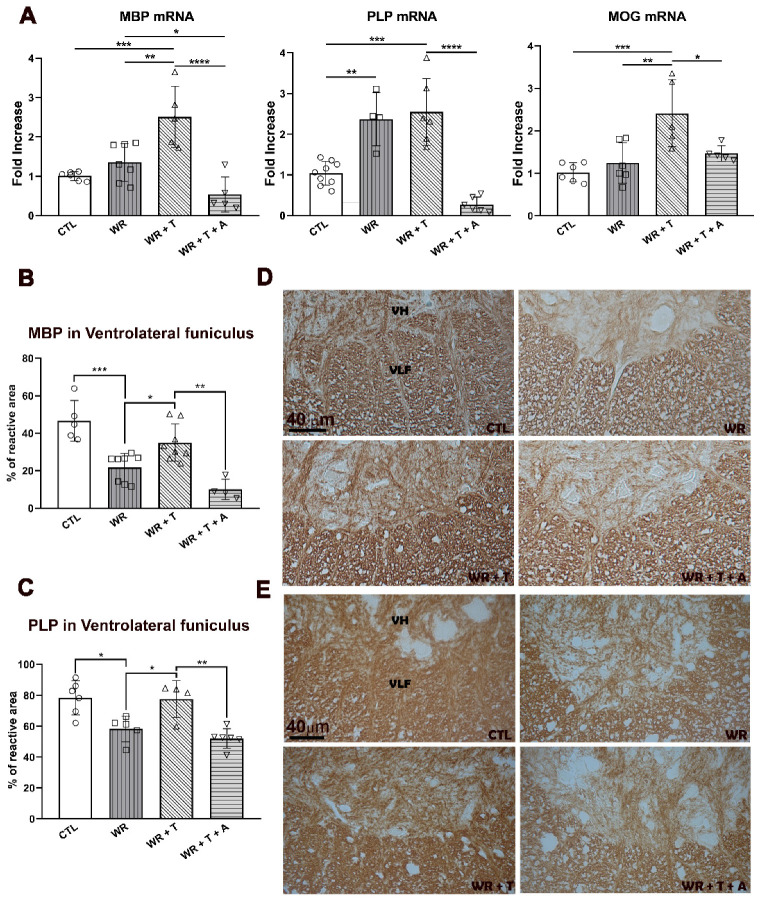
Effects of testosterone and anastrozole on the mRNA expression for the myelin proteins, MBP, PLP, and MOG (**A**) and immunoreactivity for MBP and PLP in the cervical spinal cord (**B**). (**A**) MBP mRNA (left-hand graph): CTL and *WR* shared similar expression levels, whereas the *WR* + T group showed higher values vs. control (*** *p* < 0.001) or *WR* alone (** *p* < 0.01). The *WR* + T + A group showed lower values than *WR* alone (* *p* < 0.05) and *WR* + T **** (*p* < 0.0001). PLP mRNA (middle graph): *WR* and *WR* + T groups showed increased expression vs. control (** *p* < 0.01 and *** *p* < 0.001, respectively). Aromatase inhibition (*WR* + T + A) produced a marked inhibition of PLP mRNA expression vs. *WR* + T mice (**** *p* < 0.0001). MOG mRNA (right-hand graph): CTL and *WR* showed similar mRNA levels whereas *WR* + T showed higher expression vs. control (*** *p* < 0.001) and *WR* (** *p* < 0.01). This stimulatory effect disappeared in the *WR* + T + A group (* *p* < 0.05, *WR* + T + A vs. *WR* + T). (**B**) The high immunoreactivity of MBP and (**C**) PLP in the *WR* + T group (* *p* < 0.05 vs. *WR*) was down-regulated in the ventrolateral funiculus of *WR* + T + A (** *p* < 0.01 in both cases). (**D**,**E**): Light microscope images of the ventrolateral funiculus show changes for MBP and PLP in the four groups of mice quantified in the graphs from (**B**,**C**), respectively. VH: ventral horn, VLF: ventrolateral funiculus, CTL: control, *WR*: Wobbler, T: testosterone, A: anastrozole.

ANOVA analysis showed significant group differences in MBP mRNA (F_(3,21)_ = 11.43; *p* < 0.001) in the cervical spinal cord. The multiple comparison test showed similar mRNA expression between control and *WR* mice (Figure 4A). However, MBP mRNA was higher in *WR* + T vs. untreated *WR*s (*p* < 0.01) or control mice (*p* < 0.001). However, MBP mRNA levels in the *WR* + T + A group were lower than *WR* alone (*p* < 0.05) and *WR* + T (*p* < 0.001), suggesting that T effects on MBP mRNA levels were not independent from estrogen formation.

The ANOVA test for PLP mRNA also showed significant group differences (F_(3,21)_ = 11.43; *p* < 0.001). Multiple comparisons for PLP mRNA showed a distinctive pattern. Thus, higher levels of PLP mRNA were measured in *WR* mice comparatively to control mice (Figure 4A, *p* < 0.01). T did not change PLP mRNA levels (*p* > 0.05 *WR* + T vs. *WR*). Furthermore, *WR* + T + A showed a marked reduction in PLP mRNA (Figure 4A, *p* < 0.001 vs. *WR* + T), implying estrogenic regulation of PLP expression. Finally, the ANOVA test also showed intergroup differences for MOG mRNA (F_(3,29)_ =8.271; *p* < 0.001). In resemblance to MBP, no difference was found for MOG mRNA between control and untreated *WR*s, but it was significantly increased in the *WR* + T group (*p* < 0.01 vs. empty-silastic-treated *WR*s; *p* < 0.001 vs. controls). In contrast to MBP mRNA, aromatase inhibition in T treated-*WR* mice significantly reduced MOG mRNA (*WR* + T + A: *p* < 0.05 vs. *WR* + T, Figure 4A). In conclusion, even though mRNAs for myelin proteins were not reduced in *WR*s, T treatment had a stimulatory effect on both MBP and MOG mRNAs. These results suggest a possible transcriptional effect of T on MBP, and MOG but not PLP mRNA and are in consonance with steroid effects on myelin morphology.

In the ventrolateral funiculus of the white matter, *WR*s showed a significant reduction of MBP immunoreactive protein in comparison to controls (*p* < 0.001, Figure 4B) and to *WR* + T (*p* < 0.05). Furthermore, *WR* mice showed no difference when compared to *WR* + T + A mice. Similarly, PLP immunoreaction was increased in *WR* + T vs. *WR* mice (Figure 4C, *p* < 0.05 vs. *WR*), and remained lower in *WR* + T + A vs. *WR* + T (*p* < 0.01). Figure 4D,E show representative images corresponding to MBP and PLP immunoreactive proteins. The density of mature oligodendrocytes was analyzed following detection using immunofluorescence staining with the CC1 antibody. The ANOVA test showed significant differences between experimental groups in regard to the density of cells expressed as the percentage of control values located in the ventrolateral funiculus (*p* < 0.0001, F = 33.86). A significant reduction was found in *WR*s vs. control (mean ± SD in *WR*: 61.66% ± 15.58, * *p* < 0.05 vs. control: 100 ± 6.80). *WR* + T showed a significant increase in CC1+ cell density over *WR* and control values (*WR* + T: 162.7 ± 14.76, **** *p* < 0.0001 vs. *WR* and ** *p* < 0.01 vs. controls). On the contrary, *WR* + T + A show similar values to *WR* mice (65.35 ± 19.46, NS vs. *WR*, **** *p* < 0.0001 vs. *WR* + T and * *p* < 0.05 vs. control).

### 3.5. Testosterone Reduces the Activation of Microglia and the Expression of Proinflammatory Mediators in the Wobbler Spinal Cord

**Figure 5:**
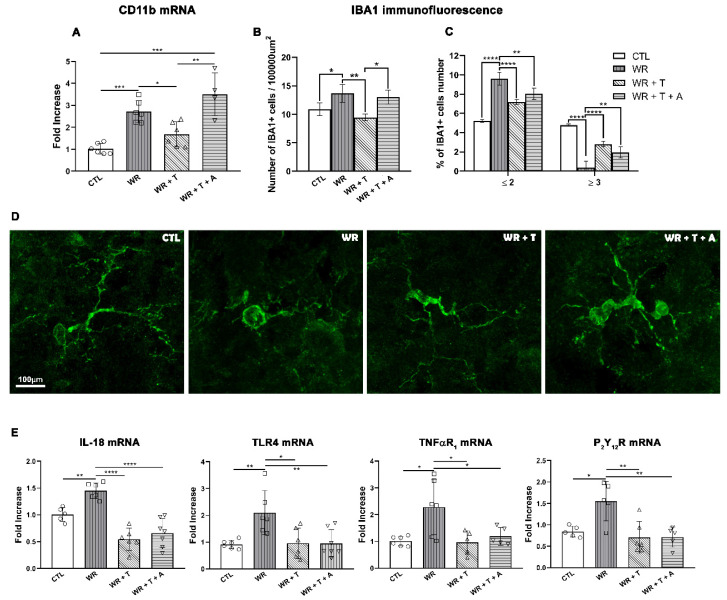
Effect of testosterone and anastrozole on inflammatory-related factors in Wobbler mice. (**A**,**B**) Androgen treatment reduced the high levels of *CD11*b mRNA and IBA1 cell density (expressed as number of IBA1+ cells/100,000 µm^2^) in *WR*s (*CD11*b mRNA: * *p* < 0.05; IBA1+ cell density: ** *p* < 0.01, *WR* + T vs. *WR*, respectively) and this effect was not observed in *WR* + T + A (*CD11b* mRNA and IBA1+ cell density: NS vs. *WR)* which showed higher levels of *CD11b* mRNA than CTL (*** *p* < 0.001). (**C**) *WR* + T and *WR* + T + A showed a lower percentage of IBA1+ cells with a low number of branches (≤2) and increased those with ≥3 branches. (**D**) Representative images of IBA1+ cells in control, *WR*, *WR* + T and *WR* + T + A in the white matter of the cervical spinal cord. (**E**) The high levels of mRNA of inflammatory factors (*IL-18, TLR4, TNFαR_1_, P_2_Y_12_R*) of untreated *WR*s were decreased in *WR* + T (**** *p* < 0.0001 for *IL-18,* * *p* < 0.05 for *TLR4* and *TNFαR_1_* and ** *p* < 0.01 for P_2_Y_12_R vs. *WR*) or in *WR* + T + A (**** *p* < 0.0001 for IL18, ** *p* < 0.01 for *TLR4*, * *p* < 0.05 for *TNFαR*_1_, ** *p* < 0.01 for *P_2_Y_12_R* vs. *WR*, respectively) in the whole cervical spinal cord. CTL: control, *WR*: Wobbler, *WR* + T: Wobbler + testosterone, *WR* + T + A: Wobbler + testosterone + anastrozole.

Proinflammatory factors produced by reactive glial cells play an important role in demyelination [46,47]. Here, we studied the expression of two markers of microglia, namely CD11b mRNA and IBA1 immunoreactive protein together with the mRNA expression of proinflammatory factors: IL-18 (an inflammatory interleukin), TLR4 (receptor for alarmins), TNFαR_1_ (a major inflammatory mediator) and the purinergic receptor (P_2_Y_12_R, signal for inflammation). ANOVA analysis showed significant group differences for CD11b mRNA (F_(3, 18)_ = 18.09, *p* < 0.0001). Post hoc comparisons revealed increased mRNA expression of CD11b in *WR* mice (*p* < 0.001 vs. CTL), which was decreased by T administration (*p* < 0.05). The T negative effect on CD11b was blunted by aromatase inhibition (*p* < 0.01 *WR* + T + A vs. *WR* + T, Figure 5A). As shown in Figure 5B, the ventral region (ventral horn of the gray matter plus ventrolateral funiculus of the white matter) from male *WR* mice displayed a significantly higher density of IBA1+ cells/area in comparison to controls (*p* < 0.05). In contrast, *WR* + T showed reduced IBA1 immunostaining comparatively to untreated *WR* mice (*p* < 0.01). The density of IBA1+ cells in *WR* + T + A was higher than in *WR* + T mice (*p* < 0.05) and was similar to empty-silastic-treated *WR*s, suggesting a role for estrogens in the T effect. Regarding their morphology, a high percentage of IBA1+ cells displayed a reactive phenotype (Figure 5C,D: cells with ≤2 branches), while *WR* + T showed cells with a higher number of branches (≥3 branches, *p* < 0.0001 vs. *WR*), characteristic of a less reactive phenotype (Figure 5C,D). High branching was also observed in the *WR* + T + A group (Figure 5C, *p* < 0.01 vs. *WR* and Figure 5D, right-hand image).

IL-18 mRNA expression in the cervical spinal cord showed significant group differences in the ANOVA test (F_(3, 20)_ = 24.54, *p* < 0.0001). The post hoc test revealed that untreated *WR*s presented higher levels of IL-18 mRNA than control mice (*p* < 0.01), which were decreased in *WR* + T (*p* < 0.0001 vs. *WR*). The cotreatment of *WR* with T + A did not modify the low levels of this interleukin comparatively to mice receiving only T (NS, *WR* + T vs. *WR* + T + A, Figure 5E). Similar results were obtained in the TLR4 mRNA via ANOVA analysis (F_(3, 21)_ = 6.320, *p* < 0.01). Tuckey’s multiple comparison test for TLR4 mRNA showed higher values in *WR* mice (*p* < 0.01 vs. control) which declined following T treatment (*p* < 0.05 vs. *WR*). Similar to results with IL18 mRNA, TLR4 mRNA showed no changes in *WR* + T + A vs. *WR* + T and displayed lower levels than untreated *WR*s (*p* < 0.01, Figure 5E).

ANOVA analysis also showed significant group differences for TNFαR_1_ mRNA (F_(3,18)_= 6.025, *p* < 0.001) and for P_2_Y_12_R mRNA (F_(3,17)_ = 7.765, *p* < 0.001). These two inflammatory mediators showed similar expression profiles in response to the androgenic steroid or anti-aromatase treatment. As shown in Figure 5E, the mRNA of TNFαR_1_ was elevated in *WR* mice compared to control mice (*p* < 0.05) and returned to control levels in *WR* + T or *WR* + T + A groups (*p* < 0.05). Post hoc analysis of the purinergic receptor showed increased P_2_Y_12_R mRNA in the *WR* group (*p* < 0.05 vs. control), which was down-regulated in both the *WR* + T and the *WR* + T + A groups (*p* < 0.01 vs. *WR*). Therefore, these four inflammatory mediators showed a similar expression profile: high expression in *WR* mice, reduction after treatment with T alone or with T plus the anti-aromatase inhibitor.

### 3.6. Effects of Testosterone and Anastrozole on Glutamine Synthetase in the Cervical Spinal Cord

**Figure 6:**
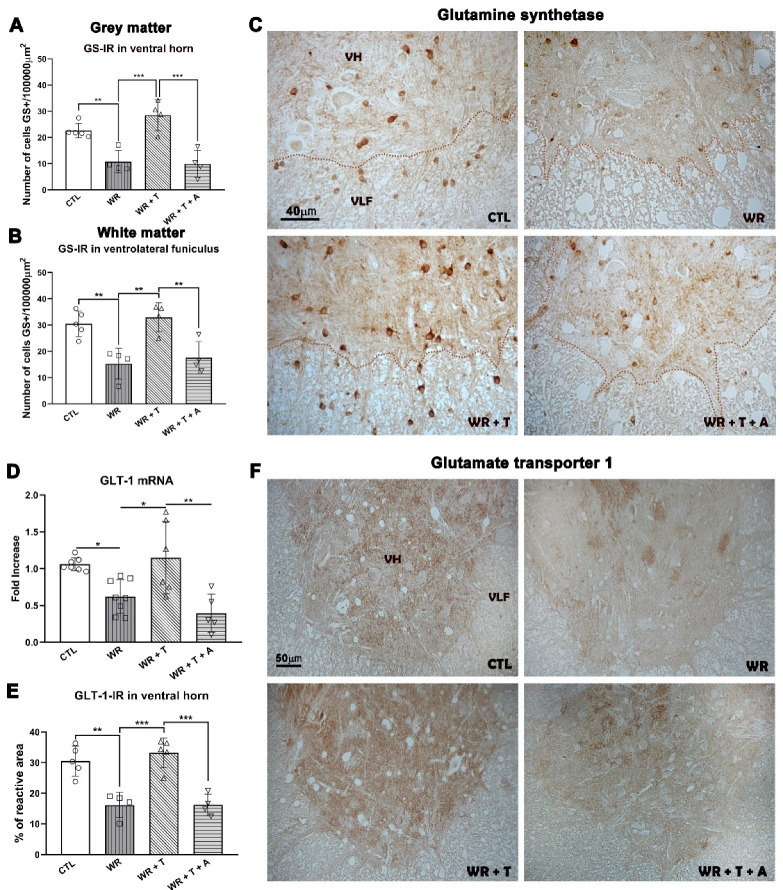
The low levels of GS and GLT-1 in Wobblers were up-regulated by testosterone treatment but not by cotreatment of testosterone plus the aromatase inhibitor. (**A**,**B**): The density of GS+ cells/area was low in *WR* and *WR* + T + A, but it increased in *WR* + T in ventral horn ((**A**), gray matter, *** *p* < 0.001) and ventrolateral funiculus ((**B**), white matter, ** *p* < 0.01). (**C**) Representative images of GS+ cells in gray (VH) and white matter (VLF) of the ventral region from the cervical spinal cord in the four experimental groups quantified in A and B. (**D**) Low expression of *GLT-1* mRNA in *WR* and *WR* + T + A compared to higher levels of *GLT-1* mRNA in *WR* + T. (**E**) Percentage of immunoreactive area was lower in the Lamina IX of *WR*s and *WR* + T + A, whereas higher percentage of immunoreactive area was shown in *WR* + T. (**F**) Representative images of GLT-1 staining in the ventral horn of the four experimental groups quantified in the graphs of (**D**,**E**). CTL: control, *WR*: Wobbler, *WR* + T: Wobbler + testosterone. *WR* + T + A: Wobbler + testosterone + anastrozole, GS: glutamine synthetase, GLT-1: glutamate trasnporter-1, VH: Ventral horn, VLF: Ventrolateral funiculus *: *p* < 0.05, ** *p* < 0.01 and *** *p* < 0.001.

Astrocytes and oligodendrocytes express GS, an enzyme that converts glutamate into glutamine in the CNS [40]. In particular, *WR* astrocytes show low expression of GS [42,48]. To study the effects of T and A on the glutamatergic system, we measured the levels of GS and the glutamate transporter GLT-1 that clears excess glutamate from synapses. In accordance with previous findings [48], *WR*s showed fewer GS+ cells/area than control mice in the ventral horn of gray matter (*p* < 0.01, Figure 6A) and the ventrolateral funiculus of white matter (*p* < 0.01, Figure 6B), while GS immunostaining returned to control levels in both regions of *WR* + T (ventral horn: *p* < 0.001 and ventrolateral funiculus: *p* < 0.01 vs. *WR*s, Figure 6A,B). However, the stimulatory effect disappeared in both regions in *WR* + T + A (*p* < 0.001 and *p* < 0.01 vs. *WR* + T, respectively, Figure 6A,B), showing similar values to empty-silastic-treated *WR*s. Figure 6C depicts representative images of GS immunostaining of the four experimental groups.

ANOVA analysis also showed significant group differences in the transporter GLT-1 mRNA (F_(3, 22)_ = 8.784, *p* < 0.001). The multiple comparison test showed a significant reduction in GLT-1 mRNA in *WR*s bearing empty silastic tubes (*p* < 0.05 vs. control, Figure 6D). GLT-1 mRNA increased after T treatment (*p* < 0.05 vs. *WR*s), an effect reversed by A (*p* < 0.01 vs. *WR* + T, Figure 6D). Similar results were observed for GLT-1 immunoreactivity in the lamina IX of the ventral horn. *WR*s showed a significant reduction in the percentage of GLT-1-IR area in comparison to controls (*p* < 0.01, Figure 6E) which was increased by T treatment (*p* < 0.001 vs. *WR*s). However, T plus A-treated *WR*s showed levels of GLT-1 immunoreaction similar to those of empty-silastic-treated *WR*s (NS vs. *WR*s, *p* < 0.01 vs. *WR* + T, Figure 6E). Figure 6F shows the representative images of GLT-1 immunoreaction in the Lamina IX of the ventral horn.

**Figure 7:**
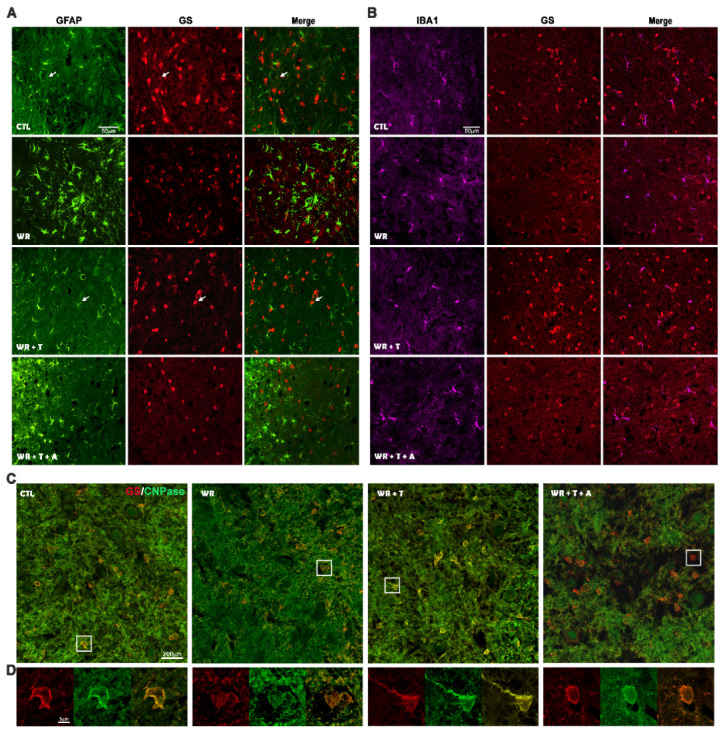
Representative images of double immunofluorescence for GS/GFAP (**A**), GS/IBA1 (**B**) and GS/CNPase (**C**,**D**) in the ventral horn (gray matter) of the cervical spinal cord from control, untreated *WR*, *WR* + T and *WR* + T + A. Arrows show cells that colocalize for GS/GFAP. The square in (**C**) is illustrated at high magnification in (**D**).

To identify the cell type expressing GS, we performed double immunofluorescence for GS^+^/GFAP^+^ (astrocytes), GS^+^/IBA1^+^ (microglia) and GS^+^/CNPase^+^ (oligodendrocytes) in cervical spinal cord sections. In all groups, double labeled GS^+^/GFAP^+^ cells showed a low level of colocalization (2–3% of cells, arrows, Figure 7A). With regard to GFAP^+^ cells (Figure 7A, green channel), empty-silastic-treated *WR*s showed a high density of GFAP+ cells in the ventral horn in agreement with previous results [30,48,49]. *WR* + T revealed low levels of immunoreaction (Figure 7A) as previously shown in Lara et al., 2021 [29]. In *WR* + T + A, the addition of the aromatase inhibitor slightly reduced this parameter. Regarding GS^+^/IBA1^+^ colocalization, no double labeled cells were found in the four experimental groups (Figure 7B). In particular, empty-silastic-treated *WR*s and *WR* + T + A showed lower immunoreactivity for GS (red channel, Figure 7A,B) and higher immunofluorescence for IBA1 than control and *WR* + T groups (Figure 7B). Double immunofluorescence for GS/CNPase revealed high levels of colocalization in controls and treated or untreated *WR*s (Figure 7C, low magnification images). Observations made at high magnification and resolution using the high-sensitivity system with a detector (Airyscan) showed lower immunofluorescence for both markers in *WR* and *WR* + T + A groups (Figure 7D).

### 3.7. Effects of Testosterone and Anastrozole on the Biceps Weight, Body Weight, Clinical Parameters and Endocrine Glands

**Figure 8:**
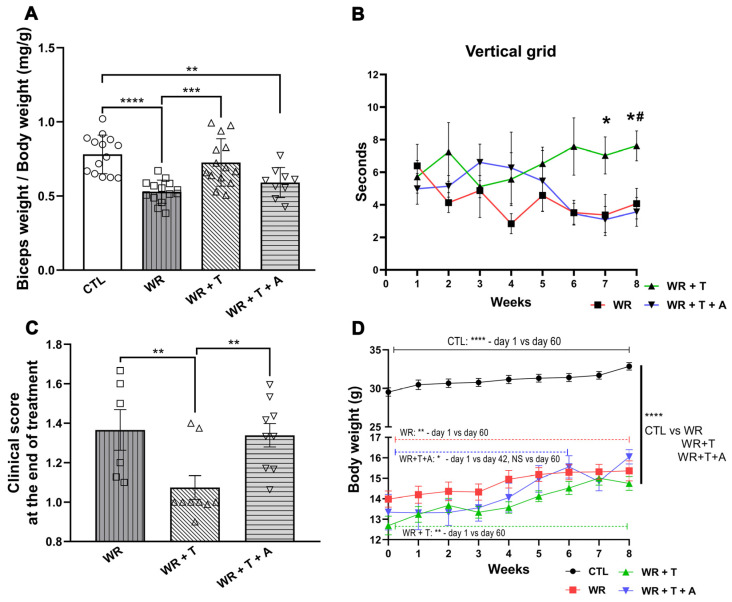
Increase in biceps muscle weight, vertical grid performance and body weight associated with low clinical score in *WR* + T but not in *WR* + T + A or *WR*. (**A**) Reduction of biceps weight/body weight in *WR*s and *WR* + T + A (*** *p* < 0.001, ** *p* < 0.01 vs. control, respectively), but not in *WR* + T mice (NS vs. control, *** *p* < 0.001 vs. *WR*). (**B**) Better performance in Vertical Grid of *WR* + T (* *p* < 0.05 vs. *WR* and # *p* < 0.05 vs. *WR* +T + A) and (**C**) lower clinical disease index score (** *p* < 0.01) vs. *WR*s and *WR* + T + A. (**D**) Control showed higher body weight than *WR*, *WR* + T or *WR* + T + A groups (**** *p* < 0.0001). Repeated measures ANOVA revealed that body weight at the end of experiment is higher than day 1 (**** *p* < 0.0001) in CTL (** *p* < 0.01) *WR* or *WR* + T, whereas *WR* + T + A group showed an increase between day 1 and day 42 (* *p* < 0.05) but not between day 1 and day 60. CTL: control, *WR*: Wobbler, *WR* + T: Wobbler + testosterone. *WR* + T + A: Wobbler + testosterone + anastrozole.

In addition to the T effects on myelination, glial reactivity, inflammatory markers and glutamatergic system described in *WR* spinal cord, chronic T administration also modified biceps weight and clinical parameters. *WR*s showed decreased biceps weight probably due to poor innervation from the ailing motoneurons (*p* < 0.0001 vs. control mice, Figure 8A). This effect was attenuated by T (*p* < 0.001), but the beneficial effects of T were not observed in the presence of A. These observations indicate the functional consequences of the coadministration of the male sex steroid with the aromatase inhibitor. Repeated measures two-way ANOVA and the Tukey post hoc test demonstrated that *WR* + T performed better in a vertical grid (*p* < 0.05, Figure 8B) whereas empty-silastic-treated *WR*s and those receiving T + A showed a similar performance after 8 weeks of treatment (Figure 8B). Moreover, *WR* + T also showed a better clinical index score at the time of sacrifice in comparison to empty-silastic-treated *WR*s and *WR* + T + A (*p* < 0.01, Figure 8C). As expected, the body weight of controls was greater than any of the *WR* groups (*p* < 0.0001, Figure 8D). There was a steady increase in body weight in controls (*p* < 0.001), *WR*s (*p* < 0.01) and *WR* + T (*p* < 0.001) for the 60 days of follow-up as shown by repetitive measures two-way ANOVA (F_(24, 505)_ = 1567, *p* < 0.05, Figure 8D), and between day 1 and day 42 in *WR* + T + A (*p* < 0.05, Figure 8D).

**Figure 9:**
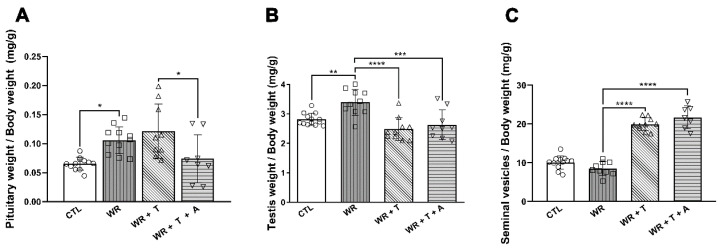
Increased size of the pituitary gland/body weight ((**A**), * *p* < 0.05) and testis/body weight in *WR* ((**B**): ** *p* < 0.01 vs. control), but only pituitary gland did not show an effect in *WR* + T (NS vs. *WR*). *WR* + T + A showed a reduced pituitary gland size compared to that of *WR* + T or *WR* ((**A**): * *p* < 0.05). *WR* + T showed a lessened size of testis/body weight ((**B**): **** *p* < 0.0001) and an increased size of seminal vesicles ((**C**): *** *p* < 0.001 vs. *WR*). No alteration of testis or seminal vesicles size was shown in the *WR* + T + A group (**B**,**C**). CTL: control, *WR*: Wobbler, *WR* + T: Wobbler + testosterone, *WR* + T + A: Wobbler + testosterone + anastrozole.

With regard to sex steroid target tissues, empty-silastic-treated *WR*s showed an elevation in pituitary/body weight and testis/body weight (* *p* < 0.05 and ** *p* < 0.01 vs. control mice, respectively, Figure 9 A,B), without changes in seminal vesicles/body weight. *WR* + T showed a decreased testis size (**** *p* < 0.001 vs. *WR*s, Figure 9B) and a trophic effect on seminal vesicles (**** *p* < 0.001 vs. *WR*s, Figure 9C), without changes in the pituitary/body weight (NS vs. *WR*s; Figure 9A). Aromatase inhibition in *WR* + T + A did not change testis or seminal vesicles mass (Figure 9B,C) but significantly decreased pituitary weight/body weight (Figure 9A: *p* < 0.05 vs. *WR* + T). Although T-derived estrogens did not apparently play a role in the effect of T in testis or seminal vesicles, the elevation of the pituitary weight in untreated male *WR*s suggests the participation of endogenous estrogens in this effect.

## 4. Discussion

The present study demonstrated several myelin abnormalities in the spinal cord of mutant *WR* mice which were mostly ameliorated by T treatment. First, in *WR* mice, myelin anomalies and low density of CC1+ mature oligodendrocytes coexisted with high levels of proinflammatory factors associated with a low expression of glutamate detoxifying factors. Neuroinflammation and glutamate toxicity were already reported in previous publications [50]. In addition, we investigated the role of T as a potential replacement therapy, considering that male *WR* mice suffer from hypotestosteronaemia and infertility [10]. We demonstrated that in *WR* mice, chronic T administration showed myelin-protective and anti-inflammatory effects. These effects may be due to normalization of T and 5α-DHT to physiological levels although for several parameters, estradiol synthesis plays an important role, as revealed by the use of an aromatase inhibitor. T treatment raised factors such as GS and GLT-1 that favor the glutamate/glutamine cycle, detoxify ammonia and glutamate from the synaptic cleft and also provide energy to neurons via oligodendrocytes and astrocytes [39]. In T-treated *WR*s, the rise in glutamine pool in the spinal cord, possibly enhances transmission efficacy acting as an energy substrate for axons. Lastly, the regulation of these factors may also depend on estradiol synthesis because *WR* + T + A lost these protective effects on the glutamate circuit.

Abnormalities of myelin sheaths were demonstrated via different procedures that include low staining of total myelin lipids with LFB and decreased density of myelinated axons assessed by using toluidine-blue-stained semithin sections and electron microscopy [51]. The latter procedure showed aberrant features of myelin with irregular, instead of circularly shaped axonal profiles, and detachment and broken myelin lamellae. Both the g-ratios and regression analysis sustained that myelin abnormalities of empty-silastic-treated *WR*s could be restored to normal via T treatment.

Because the myelin membrane contains about 15–30% protein of total dry weight, we studied if demyelination and myelin abnormalities of *WR* spinal cord are accompanied by changes in the expression of three major central myelin proteins. We found lower levels of basal MBP and PLP immunoreactivity in the cervical spinal cord of male *WR*s without an effect on their mRNAs, similarly to MOG mRNA. The low expression of MBP and PLP and the reduction of mature oligodendrocytes may be the consequence of axonal loss, neurodegeneration and neuroinflammation, findings that require further investigation. However, normal or even higher expression of myelin protein mRNA may be a compensatory mechanism to counteract the decreased levels of central myelin proteins.

Our past and present investigation of the *WR* mouse also revealed neuroinflammation with increased microglial IBA1+ cells and CD11b mRNA expression in combination with proinflammatory factors including IL18, TLR4, TNFαR_1_ and P_2_Y_12_R. These inflammatory mediators originate in activated microglia and astrocytes, which change their role from protection to pathological in *WR* mice [9,52,53]. Accordingly, TLR4 has been found to associate with other inflammatory markers, motoneuron death and low performance in tests of motor behavior [9,29,53]. TLR4 is a component of the NFkB pathway leading to cytokine production, and in this regard, this receptor induces demyelination and inflammation, as shown in diseases like multiple sclerosis [54]. The colocalization of TNFα and CD11b (a microglia marker) or GFAP (an astrocyte marker) [53,55] supports the hypothesis that pathological microglia and astrocytes of the *WR* disease participate in demyelination. Moreover, we found that IBA1+ cells in the ventrolateral funiculus of the white matter showed an activated morphology in naïve *WR*s which was reduced by T with or without A treatment. IBA1+ microglia of *WR* mice showed a round soma with low branching, a morphology suggesting reactive activation, in contrast with T-treated *WR* mice in which cells with small soma and high branching, typical of quiescent microglia, predominated. Since mRNA for inflammatory factors stayed low after T + A cotreatment, the anti-inflammatory effect of T may depend on AR or ER beta activities on microglial cells. In this regard, it is possible that 5α-androstane 3*β*,17*β*-diol, a reduced metabolite of DHT which is increased in *WR* + T [29], activates ER beta on microglial cells [56].

It is now accepted that glutamate excitotoxicity plays an important role in neurodegeneration of the *WR* mouse. Changes of the enzyme, GS, glutamate-aspartate transporter, GLAST, and GLT-1 are early findings in the spinal cord of 6-day-old postnatal and 5-month-old *WR*s [9]. In *WR*s, this alteration is not confined only to the spinal cord because increased excitatory synaptic transmission has also been found in the hippocampus [57]. Furthermore, ALS patients [58] and SOD^G93A^ presymptomatic transgenic mice also show cerebral cortex hyperexcitability, indicating that disorders of glutamate homeostasis is a common feature of ALS and its animal models [59]. Excitotoxicity follows glutamate binding to AMPA and kainate receptors causing the death of oligodendrocytes and demyelination, a mechanism reported in multiple sclerosis [60,61] that may also apply to neurodegenerative diseases. As already mentioned in the Introduction, androgens stimulate myelinogenesis and remyelination in rodents with demyelination due to cuprizone intoxication, EAE and lysolecithin treatment [18,20,62]. Furthermore, the AR shows an important role in myelin formation in males [63] and has become a promising target to stimulate remyelination [18]. In agreement, the results of the morphological, biochemical and molecular approaches of the present study suggest that T treatment restores myelination in the spinal cord of *WR* mice.

T is the main androgen circulating in mammals and produces androgenic effects upon binding to the AR present in target tissues. However, T is also a pro-hormone, because it can be metabolized to 5α-dihydrotestosterone, aromatized to estrogens and converted into 5α-androstane 3β,17β-diol (3β-diol). Both estradiol and 5α-androstane 3β,17β-diol bind to the estrogen receptor (ER) [15]. To investigate the role of estradiol in androgen neuroprotection, we administered the aromatase inhibitor, A, to *WR* mice one week before T in order to reduce the level of T aromatization. Previously, we have shown that T administration to male symptomatic *WR*s normalizes the low levels of androgens in the spinal cord to levels similar to those of control mice. However, T administration did not increase estradiol levels but slowed clinical progression of the *WR* disease [10,29]. These observations suggest that the restoration of the physiological levels of androgens is required for neuroprotection. In this work, the administration of an aromatase inhibitor in combination with T halted the improvement of clinical and molecular parameters. These results suggest that estradiol synthesis should also be preserved for the reversal of myelin abnormalities in the *WR* spinal cord. It is also possible that basal estrogen levels are necessary for enabling axonal remyelination in the white matter and preserving cellular antioxidant activity in the spinal cord. On the other hand, an accelerated clinical deterioration of *WR* + T + A triggered by the earlier inhibition of aromatase activity might not be discarded. Therefore, both androgens and estrogens are required for neuroprotection in the *WR* spinal cord. Specifically, after the comparison of results involving *WR*s receiving T alone or T + A, we found that (a) morphological changes of myelin revealed by LFB and toluidine blue staining, g-ratio analyses, and electron microscopy, (b) myelin proteins at the mRNA (MBP, PLP and MOG) and immunoreactive levels (MBP and PLP), (c) microglia markers (IBA1 and CD11b) as well as (d) glutamatergic parameters (GS, GLT1) were sensitive to the aromatase inhibition by A (Table 2). On the other hand, the decreased expression of inflammatory factors (IL-18, TLR4, TNFαR_1_ and P_2_Y_12_R) caused by T were A-insensitive, suggesting more direct anti-inflammatory effects of T (Table 2).

ALS is a progressive disease of fatal outcome, for which current approved pharmacological treatments enhance the quality of life of or prolong the life of patients for 5–6 months only. Therefore, information produced by preclinical models becomes of utmost importance for their potential translation to clinics. Since ALS patients show high circulating plasma levels of T but low DHT concentration in the cerebrospinal fluid (CSF) [64,65], their normalization may be considered as part of a protective mechanism based on the promyelinating, anti-inflammatory and neuroprotective effects due to T itself or after conversion into reduced derivatives or estrogenic compounds. Here, we showed that T’s neuroprotective property improves motor performance, and enhances muscle mass and strength in the *WR* mouse. Furthermore, at the preclinical level, DHT administration to SOD1 transgenic mouse models reduces muscle atrophy and extends lifespan [66]. On the contrary, treatment with nandrolone, a synthetic androgen, worsens disease progression. These different data warrant further preclinical as well as human studies in ALS patients receiving androgens.

## Figures and Tables

**Table 1:** List of sequences of forward and reverse primers.

Gene	Accession Number	Forward Primer (5→3)	Reverse Primer (5→3)
*MBP*	NM_001025100	ATCCAAGTACCTGGCCACAG	CCTGTCACCGCTAAAGAAGC
*PLP*	NM_199478	CTGGCTGAGGGCTTCTACAC	GACTGACAGGTGGTCCAGGT
*MOG*	NM_010814	AAATGGCAAGGACCAAGATG	AGCAGGTGTAGCCTCCTTCA
*CD11b*	NM_008401	AAACCACAGTCCCGCAGAGA	CGTGTTCACCAGCTGGCTTA
*TLR4*	NM_021297	GGCTCCTGGCTAGGACTCTGA	TCTGATCCATGCATTGGTAGGT
*TNF_α_R*_1_	NM_011609	GCTGACCCTCTGCTCTACGAA	GCCATCCACCACAGCATACA
*IL-18*	NM_008360.2	TGCCAAAAGGAAGATGATGC	ACACAAACCCTCCCCACCTA
*P_2_Y_12_R*	NM_027571.4	TTTCAGATCCGCAGTAAATCCAA	GGCTCCAGTTTAGCATCACTA
*GLT-1*	NM_001077514	CAGTGCTGGAACTTTGCCTG	GGCTATGAAGATGGCTGCCA
*Cyclophilin B*	NM_022536	GTGGCAAGATCGAAGTGGAGAAAC	TAAAAATCAGGCCTGTGGAATGTG

**Table 2:** Overview of the effects of testosterone and anastrozole on the cervical spinal cord from male *Wobblers*.

	Location	Experimental Groups		
		Male Wobbler	Male Wobbler Testosterone	Male Wobbler Testosterone + Anastrozole
Myelin parameters				
**Luxol fast blue**	Ventrolateral funiculus	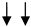	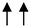	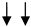
**G-ratio**	Ventrolateral funiculus	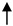	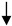	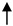
**MBP mRNA** **MBP-IR**	Whole CSC Ventrolateral funiculus	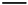 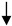	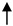 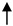	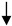 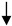
**PLP mRNA** **PLP-IR**	Whole CSC Ventrolateral funiculus	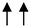 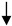	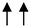 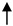	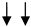 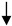
**Inflammatory factors**				
**CD11b mRNA**	Whole CSC	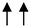	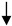	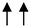
**IBA1 cell density**	Ventral region (Ventral horn + Ventrolateral funiculus)	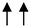	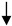	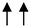
**IL-18 mRNA** **TLR4 mRNA** **TNF** **α** **R_1_ mRNA** **P_2_Y_12_R mRNA**	Whole CSC	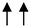	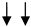	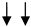
**Glutamate metabolism**				
**GS-IR**	Ventral horn Ventrolateral funiculus	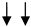 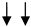	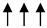 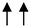	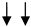 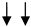
**GLT-1 mRNA** **GLT-1 IR**	Whole CSC Ventral horn	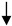 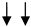	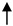 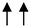	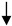 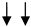

Single arrow represents slight increase (↑) or decrease effects (**↓**), whereas strong or stronger effects are represented by double (↑↑ or ↓↓) and triple arrows (↑↑↑), respectively.

## Data Availability

The data that support the findings of this study are available from the corresponding author upon reasonable request.
